# Investigating microRNAs to Explain the Link between Cholesterol Metabolism and NAFLD in Humans: A Systematic Review

**DOI:** 10.3390/nu14234946

**Published:** 2022-11-22

**Authors:** Maurice C. J. M. Konings, Sabine Baumgartner, Ronald P. Mensink, Jogchum Plat

**Affiliations:** Department of Nutrition and Movement Sciences, NUTRIM School of Nutrition and Translational Research in Metabolism, Maastricht University Medical Center, 6200 MD Maastricht, The Netherlands

**Keywords:** microRNA, cholesterol, human, NAFLD, NASH, lipoproteins

## Abstract

Non-Alcoholic Fatty Liver Disease (NAFLD) is characterized by hepatic free cholesterol accumulation. In addition, microRNAs (miRNAs) might be involved in NAFLD development. Therefore, we systematically reviewed the literature to examine the link between miRNAs and cholesterol metabolism in NAFLD. Nineteen studies were retrieved by a systematic search in September 2022. From these papers, we evaluated associations between 13 miRNAs with NAFLD and cholesterol metabolism. Additionally, their diagnostic potential was examined. Four miRNAs (miR122, 34a, 132 and 21) were associated with cholesterol metabolism and markers for NAFLD. MiR122 was upregulated in serum of NAFLD patients, increased with disease severity and correlated with HDL-C, TAG, VLDL-C, AST, ALT, ALP, lobular inflammation, hepatocellular ballooning and NAFLD score. Serum and hepatic levels also correlated. Serum and hepatic miR34a levels were increased in NAFLD, and correlated with VLDL-C and TAG. Serum miR379 was also higher in NAFLD, especially in early stages, while miR21 gave ambiguous results. The diagnostic properties of these miRNAs were comparable to those of existing biomarkers. However, serum miR122 levels appeared to be elevated before increases in ALT and AST were evident. In conclusion, miR122, miR34a, miR21 and miR132 may play a role in the development of NAFLD via effects on cholesterol metabolism. Furthermore, it needs to be explored if miRNAs 122, 34a and 379 could be used as part of a panel in addition to established biomarkers in early detection of NAFLD.

## 1. Introduction

Due to unhealthy lifestyles, non-alcoholic fatty liver disease (NAFLD)—the hepatic consequence or even cause of metabolic syndrome (MetS)—has evolved into a serious health threat [1,2,3]. The disease progression of NAFLD is a process of impairment and deterioration, which can develop from hepatic steatosis to non-alcoholic steatohepatitis (NASH), then fibrosis, further to cirrhosis and, eventually, to hepatocellular carcinoma. The first steps of this process are reversible, but structural changes remain once fibrosis has developed [4]. Over the last decades, the prevalence of NAFLD has risen fast, replacing viral hepatitis as the most prevalent liver disease, affecting almost 25% of the adult population worldwide [5].

Since NAFLD can be considered as the hepatic component of MetS, it is strongly associated with hyperlipidemia [6,7]. To explore the molecular basis of hepatic alterations in NAFLD, animal models that mimic human conditions from a physiological and metabolic point of view have provided essential insights [8]. For example, in humanized apolipoprotein E2 knock-in (APOE2ki) mice and low-density-lipoprotein (LDL) receptor-deficient mice, NASH developed after only a few days of high-fat, high-cholesterol (HFC) feeding, in which the dietary cholesterol component was essential [9,10]. Not only in NASH, but also in Niemann–Pick type C1 disease—another disease characterized by hepatic inflammation—do lipids (e.g., free cholesterol) accumulate in the liver [11]. Therefore, exploring the role of hepatic free cholesterol accumulation as part of the molecular mechanisms responsible for hepatic inflammation warrants attention.

One potential link between cholesterol metabolism and NAFLD development are so-called microRNAs (miRNAs). MiRNAs are short, non-coding RNA molecules composed of 18 to 25 nucleotides that play an important role in regulating gene expression, and are therefore involved in many crucial biological processes [12,13]. Circulating miRNAs are extremely stable and protected from RNase degradation. They have therefore emerged as attractive candidate targets for interventions and as biomarkers for the early diagnosis of diseases and for monitoring of disease progression [14]. In recent reviews, the roles of miRNAs in NAFLD or cholesterol metabolism have been discussed [15,16,17]. However, no attention was paid to the link of miRNAs with cholesterol metabolism as it related to NAFLD in humans, which is the focus of the current systematic literature review. This knowledge may contribute to the understanding of whether (dietary) interventions targeting these miRNAs can modify hepatic cholesterol metabolism and, consequently, NASH development and/or progression. In addition, due to the invasive nature of a liver biopsy and the difficulty of using specific and sensitive ultrasound-based methods to differentiate between steatosis, inflammation and fibrosis at a large scale [18,19], there is an urgent need for novel non-invasive tools to diagnose and monitor the progress of the different stages in NAFLD. Therefore, the possibility to use specific miRNAs as an NAFLD biomarker will also briefly be discussed.

## 2. Methods

### 2.1. Search Strategy

A systematic literature search in three databases (Cochrane Central Register of Clinical Trials, Embase and Medline) was conducted in September 2022 to identify potentially relevant studies. Search terms consisted of keywords related to cholesterol metabolism, miRNAs and NAFLD. The following terms were used: Cholesterol (cholesterol or LDL-cholesterol or HDL-cholesterol or chylomicron-cholesterol) and NAFLD (NAFLD or NAFL or NASH or steatohepatitis or steatosis) and micro-RNA (miRNA or microRNA) and limited to humans.

### 2.2. Selection of Studies

Human studies that examined associations between cholesterol, miRNA and NAFLD were selected. The selection procedure consisted of 2 steps. In the first step, titles and abstracts of all retrieved papers were screened for the following aspects: (1) studies on humans with NAFLD, (2) plasma/serum and/or hepatic cholesterol measured, (3) miRNA measured either in plasma/serum and/or liver, (4) original research (e.g., no reviews, conference abstracts, proceedings or case reports), (5) written in English and (6) no duplicates. The selection was performed independently by two researchers (M.C.J.M.K. and S.B.). When inconclusive, eligibility was discussed by both researchers until agreement was reached. In the second step, the papers that were selected in step 1 were read as full papers to assess final eligibility and for data collection.

### 2.3. Data Collection

Data collection was performed using an a-priori-defined spreadsheet and included publication characteristics (reference number, first author, year of publication), study and patient characteristics (ethnicity, sample size, health status, NAFLD stage and age), analytical methods used and results.

## 3. Results

**Figure 1:**
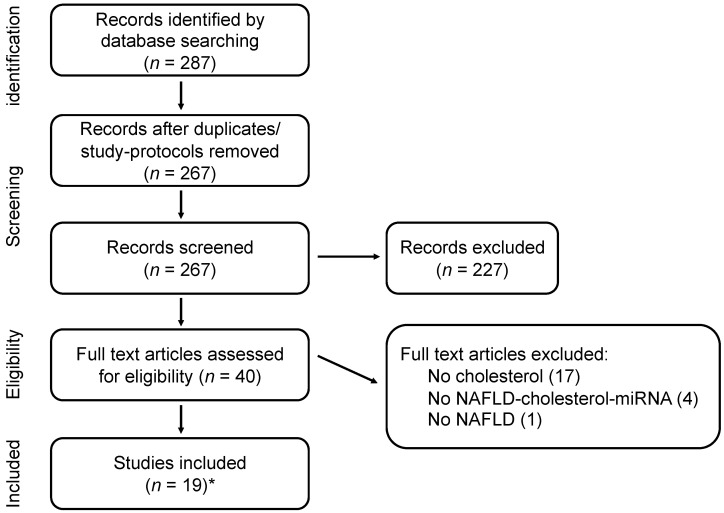
Flow chart of the study selection process. The literature search retrieved 287 potentially relevant papers; 228 were excluded after screening titles and abstracts, 40 articles were reviewed in full and ultimately 19 studies were included in the systematic review. * One study included from the reference list of the selected papers.

The systematic literature search resulted in 287 potentially relevant articles. Screening of titles and abstracts resulted in exclusion of 227 articles based on the predefined selection criteria. After reviewing the full texts of the remaining 40 articles, another 22 articles were excluded, since one or more parameters from the line of interest, i.e., miRNA, cholesterol and NAFLD, were missing. After the full selection procedure, a total of 19 studies were included. One article was retrieved from the reference list of one of the selected articles. A flowchart of the selection process is presented in Figure 1.

### 3.1. Study Characteristics and Selected miRNAs

In the 19 selected articles, 13 different miRNAs were evaluated. These 13 miRNAs (Table 1) have not been studied at the same level of detail. The most information was available for miR122 [20,21,22,23,24,25,26,27], which is one of the most abundant miRNAs in the liver and is possibly involved in hepatic disease progression; for miR34a [22,25,26,28,29], which is a critical tumor suppressor [30]; and for miR21 [22,25,26,31,32,33], which is a cancer-promoting miRNA targeting tumor-suppressor genes [34].

The remaining ten miRNAs, which were studied in less detail, were miR29a [24], miR379 [35], miR33a [21,31,36], miR33b and miR33b* [21,36], miR144 [36], miR451 [26], miR132 [37,38], miR129 [39] and miR486 [40]. MiRNA data were not uniformly expressed. For example, levels of miR122 were absolutely quantified against a calibration curve [22,24] or expressed relative to those of their controls [20,21,23,25,26,27]. MiR29a was only absolutely quantified [24], while miR34a and miR21 were absolutely quantified [22] or, like the other miRNAs, relatively expressed [20,21,23,25,26,27,28,29,31,32,33,35,36,37,39,40,41].

**Figure 2:**
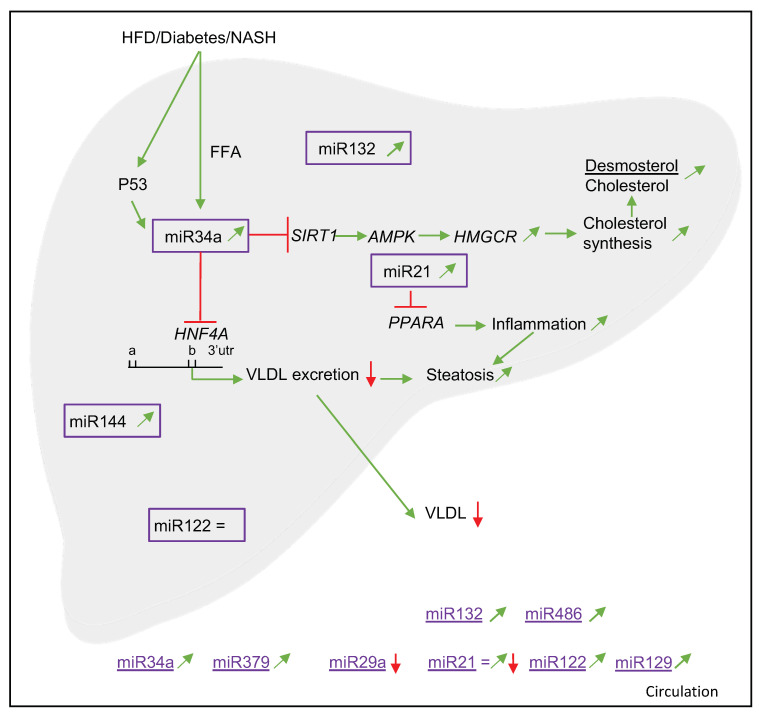
Schematic overview of potential mechanisms of miRNAs involved in cholesterol metabolism in humans. Red arrow is downregulated. Green arrow is upregulated, = is no change. HFD—high-fat diet; NASH—non-alcoholic steatohepatitis; FFA—free fatty acids; *SIRT1*—sirtuin1; *AMPK*—5′-adenosine-monophosphate-activated protein kinase; *HMGCR*—3-hydroxy-3-methylglutaryl-CoA reductase; *HNF4A*—hepatocyte nuclear factor 4 alfa; VLDL—very-low-density lipoprotein; *PPARA*—peroxisome proliferator-activated receptor alpha.

In the next paragraphs, the findings for the 13 different miRNAs have been summarized. For each miRNA, we have described their associations with cholesterol metabolism and NAFLD. When possible, additional information related to diagnostics, target genes and mechanisms has been provided. How these miRNAs may be involved in cholesterol metabolism in NAFLD have been summarized in Figure 2.

### 3.2. miR122

#### 3.2.1. Associations with NAFLD and Cholesterol Metabolism

In all eight studies that measured miR122, serum/plasma miR122 concentrations were higher in NAFLD patients as compared to their controls [20,21,22,23,24,25,26,27]. In addition, concentrations increased with disease severity [21,22,24,26]. In one study, however, levels were increased in patients with mild and moderate steatotic/fibrotic stages as compared to those of patients with more severe steatotic/fibrotic stages, but still 4-5-fold higher than those of normal controls [23]. Moreover, serum miR122 correlated negatively with serum high-density lipoprotein cholesterol (HDL-C) concentrations [21,23] and positively with serum triacylglycerol (TAG) [20,23] and very-low-density lipoprotein cholesterol (VLDL-C) concentrations [20], total cholesterol (TC), low-density lipoprotein cholesterol (LDL-C) [22], alkaline phosphatase (ALP) [21], aspartate-aminotransferase (AST) [21,22,23], alanine-aminotransferase (ALT) [21,22,23] and with the NAFLD Activity Score (NAS) [24]. More results of these studies are presented in Table 2.

**Figure 3:**
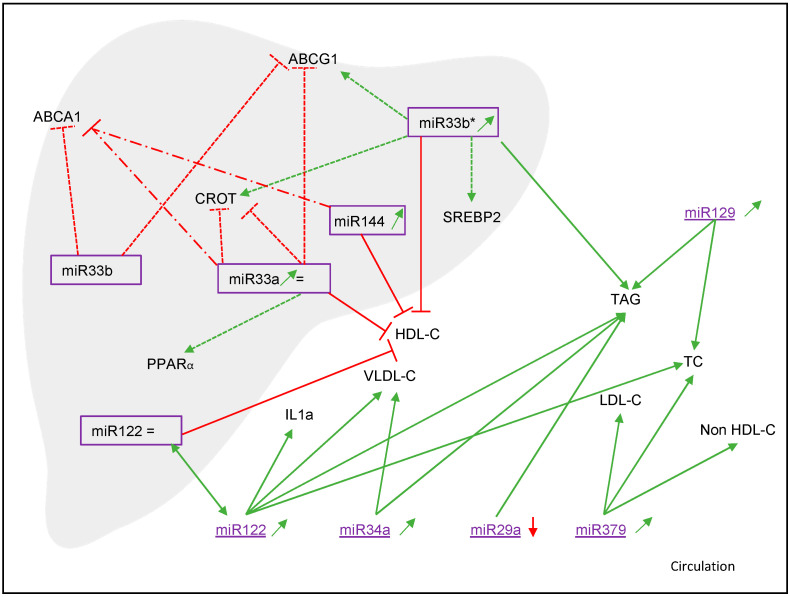
Relations between miRNAs, genes and cholesterol metabolism. Green: positive correlations; red: inverse correlations; small dotted lines: mRNA levels; broad–small dotted lines: protein levels; solid lines: other relations. Red arrow is downregulated. Green arrow is upregulated, = is no change; IL-1α—Interleukin 1 alfa; TAG—triacylglycerol; VLDL-C—very-low-density lipoprotein cholesterol; HDL-C—high-density lipoprotein cholesterol; LDL-C—low-density lipoprotein cholesterol; TC—total cholesterol; Non-HDL-C—non-high-density lipoprotein cholesterol; ABCA1—ATP-binding cassette transporter A1; ABGG1—ATP-binding cassette transporter G1; SREBP2—sterol regulatory element-binding protein 2; CROT—carnitine O-octanoyltransferase; PPAR⍺—peroxisome proliferator-activated receptor alpha.

Auguet et al. [21] found a weak but significant positive correlation between hepatic and plasma miR122 levels. Additionally, a positive correlation between serum miR122 levels and the fibrotic stage has been reported [22,24]. Moreover, a positive correlation between plasma miR122 concentrations and lobular inflammation [21,22] and hepatocellular ballooning [21] was observed. This aligns with the observation of Abdel-Hamed et al. [20], who reported in NAFLD patients a positive correlation between serum miR122 and serum levels of IL-1α (Figure 3), an inflammatory cytokine. They also found that NAFLD patients were more frequent carriers of the high-risk allele DD (rs3783553), which is an insertion/deletion polymorphism in the 3′ UTR of the IL-1α allele. This polymorphism interrupts the binding of miR122, and Abdel-Hamed et al. [20] have reported in NAFLD patients an increased relative expression of miR122 in these DD-carriers compared to that in the low-risk genotype ID or II. Finally, Yamada et al. [26] have found a positive association between serum miR122 levels and severity of steatosis in both men and women.

#### 3.2.2. Diagnostics

Receiver Operation Characteristic (ROC) curves for serum miR122 showed an Area Under the Curve (AUC) of 0.83, which means that 83% of NAFLD patients were correctly discriminated from healthy controls [24]. Both Salvazo et al. [22] and Cermelli et al. [25] showed better, or at least similar, curve characteristics (Table 3) than for ALT, suggesting that serum miR122 levels can be used as a biomarker to discriminate NAFLD patients from healthy controls. Ye et al. [27] also showed an AUROC of 0.77 for miR122. Finally, Auguet et al. [21] concluded that the accuracy of miR122 in discriminating NAFLD from non-NAFLD patients resulted in an AUROC curve of 0.82. Moreover, for distinguishing advanced disease from a mild clinical form, the diagnostic value for miR122 was 0.76. Finally, using logistic regression analysis and after adjusting for BMI, HDL-C, TAG, AST and ALT, serum miR122 levels showed the highest odds ratio (OR: 2.19) to discriminate patients with and without hepatocellular ballooning.

#### 3.2.3. Target Genes and Mechanisms

Target genes of miR122 and the other miRNAs are shown in Table 1. For hepatic miR122 expression, no correlation was observed with genes directly related to lipid metabolism [21].

### 3.3. miR34a

#### 3.3.1. Associations with NAFLD and Cholesterol Metabolism

Serum levels [22] and hepatic expression [25,26,28,29] of miR34a were significantly higher in NAFLD patients as compared to healthy controls. Moreover, serum miR34a levels in NAFLD patients were significantly increased with severity of liver steatosis [25], fibrosis and hepatic inflammation [22,25]. However, these findings were not supported by Yamada et al. [26]. In addition, a positive correlation has been found between serum miR34a and VLDL-C concentrations as well as between miR34a and TAG concentrations in NAFLD patients [25].

#### 3.3.2. Diagnostics

Salvazo et al. and Cermelli et al. [22,25] reported that the diagnostic value of miR34a to discriminate NAFLD patients from healthy controls was comparable to that of ALT (AUC = 0.78/ 0.75 vs. 0.83).

#### 3.3.3. Target Genes and Mechanisms

Hepatocyte nuclear factor 4 alfa (*HNF4A*) is a nuclear hormone receptor that controls the basal expression of many genes involved in bile acid, lipid, glucose and drug metabolism. Xu et al. [29] described that the miR34a-*HNF4A* pathway is highly active under conditions of metabolic stress such as diabetes, high-fat feeding and NASH. In addition, p53 is an oxidative-stress-inducible protein that upregulates miR34a expression in humans and also promotes liver steatosis in mice [29]. This protein was upregulated 6.7-fold in NASH patients (*p* < 0.05), which could at least partly explain the finding that serum miR34a levels were significantly higher in NAFLD patients as compared to healthy controls [25,28]. Moreover, Xu et al. [29] found that free fatty acids (FFA), cholesterol and p53 are all upstream activators for the miR34a-*HNF4A* pathway in diabetes, obesity and NASH.

In the study performed by Min et al. [28], 3-hydroxy-3-methylglutaryl-CoA reductase (*HMGCR*) phosphorylation was decreased in NAFLD and NASH patients, indicating higher activity of this enzyme [42]. Increased miR34a levels, as observed in NASH patients, suppress sirtuin 1 (*SIRT1*). This protein upregulates 5′ adenosine monophosphate-activated protein kinase (*AMPK*), a known positive HMGCR phosphorylation regulator. This suggests that miR34a activation translates into increased HMG-CoA reductase activity and higher endogenous cholesterol synthesis. The functional impact of the relatively dephosphorylated state of HMGCR in NASH patients was confirmed by a significant increase in circulating desmosterol/cholesterol ratios compared to controls (*p* < 0.01) [28].

### 3.4. miR21

#### 3.4.1. Associations with NAFLD and Cholesterol Metabolism

Rodrigues et al. [32] showed significantly higher hepatic miR21 expression in NAFLD patients with increasing levels in the progression from steatosis to NASH (*p* < 0.01). Similar results were found in serum and skeletal muscle (*p* < 0.05). Moreover, the potential of serum miR21 concentrations as a biomarker for NAFLD stage was evaluated and a significant ±3-fold (*p* < 0.05) increase in serum miR21 levels was found between patients with steatosis and NASH. Comparable results were reported by Loyer et al. [41], i.e., a 3-fold increase in liver tissue (*p* < 0.01), but only in NASH and not in simple steatotic livers. Compared to healthy controls, Yamada et al. [26] found significantly increased miR21 serum levels in male NAFLD patients (*p* < 0.01), but not in female patients. In contrast, Sun et al. [33] showed decreased serum miR21 levels in NAFLD patients compared to healthy controls (*p* < 0.05), while no differences between NAFLD patients and their controls were found [22,25]. Loyer et al. [41] found positive correlations between hepatic miR21 levels and hepatic ballooning (*p* < 0.001), lobular inflammation (*p* < 0.001), steatosis (*p* < 0.01) and fibrosis (*p* < 0.05). Lendvai et al. [31] found a positive correlation between serum miR21 with AST levels in the steatotic group, but not in hepatitis C, steatotic hepatitis C and normal liver groups.

#### 3.4.2. Diagnostics

There were no diagnostic data provided related to miR21.

#### 3.4.3. Target Genes and Mechanisms

In human hepatoma cells, *HMGCR* was a direct target of miR21 [33]. In line with these observations, HMGCR mRNA and protein levels in serum were significantly increased (*p* < 0.01 for mRNA, *p* < 0.05 for protein) in NAFLD patients as compared to healthy controls [33].

### 3.5. Other miRNAs (miR379, miR29a, miR144, miR33a/b, miR33b*, miR451, miR132, miR129 and miR486)

#### 3.5.1. Associations with NAFLD and Cholesterol Metabolism

Okamoto et al. [35] described a 3.5-fold (*p* < 0.05) increase in serum miR379 levels in a Japanese population of NAFLD patients compared to those of control patients. When patients (*n* = 79) were divided into steatotic patients (*n* = 9) and NASH patients (*n* = 70), miR379 levels were significantly higher in the steatotic group than in controls, while levels were comparable in the NASH group. However, when patients were divided based on Brunt stages, e.g., patients with NAFLD early stage (non-alcoholic fatty liver (NAFL); Brunt stages 0–1, *n* = 53) and NAFLD advanced stage (Brunt stage 2–4, *n* = 26), they found in the early stage 3.65-fold (*p* < 0.05)—and in the NAFLD population, 4.87-fold (*p* < 0.05)—higher miR379 serum levels as compared to control patients (*n* = 10). MiR379 levels correlated positively with ALP, TC, LDL-C and non-HDL-C in early stage NAFLD patients (*n* = 51). Moreover, in the non-statin-treated patients of this group (*n* = 42), a significant positive correlation between serum TC and miR379 was found.

For miR29a, serum levels were significantly lower in NAFLD patients compared to control patients (*p* < 0.01) [24]. In addition, serum miR29a levels in patients with NAS scores < 4 were significantly lower than serum levels in healthy controls (*p* < 0.05). Unexpectedly, serum miR29a positively correlated with serum TAG concentrations within the NAFLD population.

Yamada et al. [26] found increased serum levels of miR451 in Japanese NAFLD patients compared to healthy controls, but no association with disease state. Hanin et al. [37] found a 13-fold upregulation of miR132 in postmortem hepatic tissues of NAFLD patients as compared to apparently healthy controls (*p* < 0.01). Moreover, a significant downregulation of its targets *FOXO3*, *PTEN* and *SIRT1* (*p* < 0.05 for all) and a significant upregulation of *P300* (*p* < 0.05) were observed.

Vega-Badillo et al. [36] demonstrated that hepatic miR144 had significantly higher expression in NASH patients than in control patients and patients with simple steatosis (*p* < 0.05 for both). Furthermore, miR33a was elevated in the simple steatosis group [31,36] and significantly higher in the NASH individuals (*p* < 0.01) compared to control patients [36]. Wang et al. [39] showed significantly higher miR129 levels in NAFLD patients compared to controls (*p* < 0.001). Serum levels of miR486 were also significantly increased in an overweight/obese group, which included children with NAFLD [40]. Hepatic miR33b* expression was higher in morbidly obese women with NAFLD as compared to morbidly obese women with a healthy liver and a comparable BMI (*p* < 0.001) [21]. This significantly higher expression was found in morbidly obese women with NASH (*p* < 0.001) and with simple steatosis (*p* < 0.05). Compared to women with a healthy liver, miR33b* expression was upregulated in moderately obese NAFLD women (*p* < 0.05) and the expression of miR33b* was higher in NASH patients (*p* < 0.05). Furthermore, in moderately obese women, hepatic miR33b* correlated positively with hepatic ballooning (*p* < 0.005) and lobular inflammation (*p* < 0.001). Circulating miR33b* serum levels were significantly higher in moderately and morbidly obese women as compared to normal weight women (*p* < 0.001); serum miR33b* correlated positively with concentrations of TAG and AST and negatively with that of HDL-C [36]. In contrast, Vega-Badillo et al. [36] observed an inverse correlation with plasma HDL-C and miR33a, and miR144, but not miR33b, levels. Lendvai et al. [31] showed a negative correlation between hepatic miR33a and serum AST and ALP levels. Wang et al. [39] found positive correlations between miR129 with TC and TAG but not with ALT. Logistic regression analysis suggested that miR129 can be independently influenced by TC. Al Azzouny et al. [40] showed in their multivariate analysis that miR486 expression was an independent predictor for NAFLD susceptibility.

#### 3.5.2. Diagnostics

For miR379, Okamoto et al. [35] found AUROC values for diagnosing NAFLD (0.72), NAFL (0.76), NASH (0.72), early stage NAFLD (0.74) and advanced stage NAFLD (0.67). This data suggests that this miRNA would be slightly more specific in detecting early stages of NAFLD than later stages. Jampoka et al. [24] investigated miR29a as a possible biomarker to diagnose NAFLD and found an AUC of 0.68 (*p* < 0.01) with 60.9% sensitivity and 82.4% specificity. Wang et al. [39] found AUROC values for distinguishing NAFLD from healthy controls (0.93) with a sensitivity and specificity of 83.8% and 92.7%, respectively. There were no studies describing the diagnostic value of miR144, 33a, 33b and 33b*, 132 or 486.

#### 3.5.3. Target Genes and Mechanisms

Okamoto et al. [35] identified 1423 potential target genes of miR379, which were classified according their function association or related biological processes according to Gene Ontology (GO) terms. Of all processes, biological regulation, metabolic and cellular processes covered over 70% of all pathways. After a more selective process, 27 genes were selected, which were grouped into four categories (Table 1). These categories were: fibrosis and inflammation (8 genes), energy management including lipogenesis (12 genes), cell survival and proliferation (6 genes), and cell signaling (1 gene).

Jampoka et al. [24] predicted the target genes of miR29a, as presented in Table 1. Vega-Badillo et al. [36] showed a significant inverse correlation between miR33a and hepatic ABCA1 mRNA (*p* = 0.050) and hepatic ABCA1 protein levels (*p* < 0.01). Moreover, miR33b inversely correlated with both hepatic ABCA1 mRNA (*p* < 0.001) and ABCG1 mRNA (*p* < 0.005). For miR144, an inverse correlation with hepatic ABCA1 protein (*p* < 0.05) was found. Auguet et al. [21] found positive correlations between miR33b* and *SREBP2* (*p* < 0.005) and *ABCG1* (*p* < 0.01), genes related to lipoprotein secretion. They also found positive associations for hepatic miR33a with *PPARA* (*p* < 0.05) expression and negative associations between hepatic miR33a and *ABCG1* (*p* < 0.01) expression.

### 3.6. Animal Data

**Figure 4:**
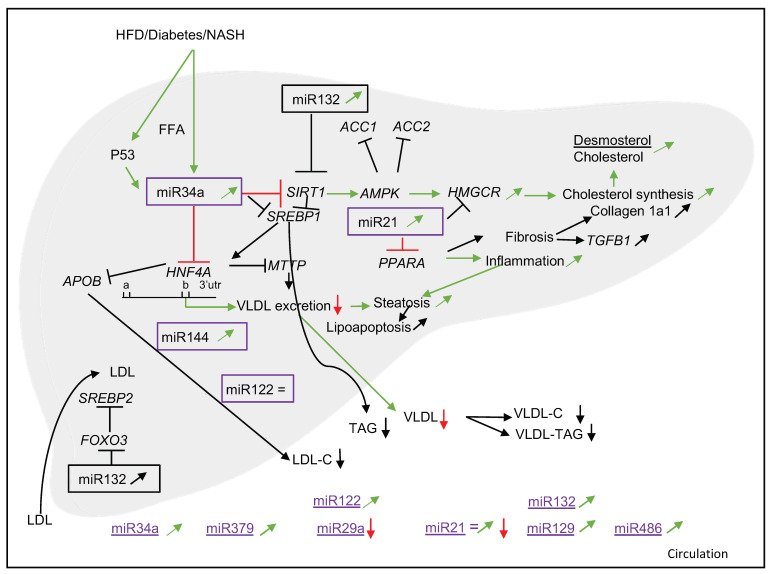
Schematic overview of potential mechanisms in animals (black) of miRNAs involved in cholesterol metabolism. Red arrow is downregulated. Green arrow is upregulated, = is no change. Data are derived as published in the papers selected for this review. HFD—high-fat diet; NASH—non-alcoholic steatohepatitis; FFA—free fatty acids; *SIRT1*—sirtuin1; *AMPK*—5′-adenosine-monophosphate-activated protein kinase; *HMGCR*—3-hydroxy-3-methylglutaryl-CoA-reductase, *HNF4A*—hepatocyte nuclear factor 4 alfa; VLDL-C—very-low-density lipoprotein cholesterol; LDL-C—low-density lipoprotein cholesterol; *PPARA*—peroxisome proliferator-activated receptor alpha; *ACC1/2*—Acetyl-CoA carboxylase1/2; *TGFB1*—transforming growth factor beta 1; *APOB*—apolipoprotein B; *MTTP*—microsomal triglyceride transfer protein; *SREBP1/2*—Sterol regulatory element-binding protein 1/2; *FOXO3*—forkhead-box protein O3.

Other than the human data, we also examined the animal data that were presented in the 19 selected articles (Figure 4). This figure shows what is mechanistically known from the animal data present in the selected papers and is useful to compare the overlap with the human condition. The animal data will be further used in the discussion.

## 4. Discussion

In this systematic review, we identified 13 miRNAs that may be associated with both NASH and cholesterol metabolism. It was suggested that four of these miRNAs (miR122, miR34a, miR21 and miR132) play a role in the development of NASH through effects on cholesterol metabolism. Therefore, it might be interesting to explore whether (dietary) interventions targeting these miRNAs can modify cholesterol metabolism, and hence, NASH development and/or progression. For the other nine miRNAs, the data was less conclusive. Furthermore, miR122, miR34a and miR379 may, in addition to traditional markers, be used in a panel for the early detection of NAFLD and/or monitoring of NAFLD development.

The most frequently studied miRNA that appeared in our literature search was miR122. In serum, miR122 concentrations were higher in patients with liver steatosis as compared to healthy controls, and concentrations were even higher in NASH patients [20,21,24,25,26,43]. Interestingly, Cheung et al. [44] showed lower hepatic miR122 expression in simple steatosis and NASH patients as compared to healthy controls, which resulted in an inverse correlation between liver and plasma levels of miR122 in NASH patients. This contrasts the findings of Auguet et al. [21], who demonstrated higher miR122 levels in morbidly obese patients with NASH compared to morbidly obese patients with simple steatosis and morbidly obese patients with normal livers. Studies in primary mouse hepatocytes and mouse models showed that phosphomevalonate-kinase (*PMVK*)—which catalyzes phosphorylation of mevalonate [45]—together with *HMGCS1*, *HMGCR* and *DHCR7* were target genes of miR122. This suggest that miR122 is involved in the regulation of cholesterol and fatty acid metabolism [46].

Another miRNA, which according to our search may be linked to both cholesterol metabolism and NAFLD, is miR34a. This miRNA is found in high levels in plasma and the liver, especially in patients with steatosis and NASH [47,48]. Furthermore, Xu et al. [29] found that upregulation of miR34a inhibits hepatic VLDL secretion, thereby promoting steatosis in which a role for *HNF4A* was postulated. Moreover, in rodents, the p53/miR34a/*SIRT1* pathway—or so-called proapoptotic pathway—is prevented by treatment with ursodeoxycholic acid (UDCA), which downregulates miR34a, in a cell-type-specific mechanism [47]. This suggests that inhibition of miR34a could prevent disease progression. Moreover, Ding et al. [49] showed in human cell lines and mice models not only that *PPARA*, its downstream genes and *SIRT1* are targets of miR34a but also that inhibition of miR34a decreased steatosis.

MiR21 was the third miRNA that appeared from our search. Rodrigues et al. [32] reported that miR21-knock-out mice—as compared to wild-type mice—who were fed a Western-type diet supplemented with obeticholic acid showed minimal steatosis, inflammation and lipo-apoptosis, which was most likely caused by an upregulation of *PPARA* and Farnesoid-X-Receptor (*FXR*) activation. In line with this, Loyer et al. [41] found that either knocking out miR21 or using an miR21 antagonist decreased liver injury, inflammation and fibrosis in mice. Moreover, they also reported increased hepatic expression of miR21 in NASH patients but not in steatotic patients, predominantly in inflammatory and biliary cells. Zhao et al. [50] showed that overexpression of miR21 stimulated extracellular-signal-related kinase1 (*ERK1*) signaling and epithelial–mesenchymal transition (EMT) of hepatocytes by targeting sprouty2 (*SPRY2*), which is a negative feedback regulator of multiple receptor kinases and *HNF4A* [50]. Changing this negative feedback mechanism may affect hepatic fibrosis development in NASH [50]. Although hepatic levels of miR21 were consistently higher in NASH and cirrhotic livers, differences in miR21 plasma or serum values were less consistent.

The fourth and final miRNA that we described was miR132. Although human data is limited, there are indications that hepatic miR132 expression is upregulated in liver biopsies from NAFLD patients compared to apparently healthy controls [37]. In mice, Hanin et al. [37] investigated the role of the direct target genes of miR132, which are involved in several steps of lipid and cholesterol metabolism. They concluded that the development of hepatic steatosis may be the consequence of affecting the various miR132 targets simultaneously when miR132 is elevated. Moreover, Zong et al. [38] found in the non-T2DM subgroup positive associations between serum miR132 and ALT, TAG, apoE and NAFLD.

Liver biopsies are the gold standard to diagnose the presence of NAFLD—more specifically, of NASH—and to monitor disease progression over time [51]. However, taking liver biopsies is not without risk, and there is a strong interest for other approaches to diagnose and monitor NASH development. Although different non-invasive imaging techniques and plasma biomarkers such as ALT and AST have been used, most patients with NAFLD remain asymptomatic and have non-elevated ALT levels [52]. Therefore, additional measurements such as plasma glucose, TAG, TC and lipoprotein cholesterol levels, in combination with BMI, fat distribution and family history [53,54] are frequently included to diagnose NAFLD. We here also explored whether the identified miRNAs could have an added value for diagnosing and monitoring NAFLD or its sub-stages. Our data suggests that three of the thirteen identified miRNAs could qualify for this purpose. Yamada et al. [26] found a positive correlation between steatosis severity and serum miR122 levels, suggesting that serum miR122 levels could be used to differentiate between healthy and diseased conditions. Jampoka et al. [24] also showed that serum miR122 levels were lower in patients with a NAFLD Activity Score (NAS) < 4 as compared to patients with a score > 4. Later, Ye et al. [27] confirmed the results of Auguet et al. [21] and Jampoka et al. [24] that miR122 levels are a more sensitive predictor than traditional serological markers for NAFLD. The question is whether we should rely on elevated miR122 levels only. It might be more attractive to include miR122 levels as part of a panel of different biomarkers to improve the accuracy of a non-invasive diagnosis of NAFLD [21]. Indeed, Ye et al. [27] developed a statistical model in which the traditional parameters such as TG, LDL-C, ALT and AST showed no significant change from baseline values until 4 weeks after onset of the disease. When miR122 was included in the model, it was possible to detect changes from week 1 after onset. Besides miR122, miR379 is another possible biomarker for diagnosing NAFLD. This miRNA had good AUROC values for all stages with slightly better performance for the earlier stages. However, a direct comparison of miR379 with more traditional biomarkers, or the added value when miR379 is included to a panel of traditional markers, is lacking. Finally, a third possible candidate miRNA that appeared from our search was miR34a. As for the other two miRNAs, plasma levels are increased with disease severity [47]. Cermelli et al. [22] already suggested that miR34a, as well as miR122, may represent novel, noninvasive biomarkers for diagnosis and histologically confirmed disease severity in patients with NAFLD. Pillai et al. [55] showed AUROCS of >0.80 between the plasma levels of five miRNAs (122, 34a, 375, 21 and 16) and three blood markers, and a positive correlation with different stages of metabolic dysregulation. Harrison et al. [56] even concluded that their NIS4 panel which consisted of miR34a and three other blood biomarkers has potential to reduce unnecessary liver biopsies in patients with lower risk of disease progression. Based on these findings, we suggest not to focus on one particular miRNA, but rather, to explore whether one or more of the identified miRNAs 122, 379 and 34a could be added to the traditional biomarkers to diagnose the presence and severity of NAFLD. Of course, this suggestion should be validated in future studies.

Compared with the relatively limited number of human studies using human samples, many more experiments have been carried out in animals. Data from animal studies that were part of the papers selected in our systematic literature search were therefore used to further explore possible underlying mechanisms to identify potential leads for future human research (Figure 4). For example, Xu et al. [29] showed that *HNF4A* was downregulated by miR34a in several mouse models and in human HepG2 cells. This consistency between species could relate to the fact that 3′-UTR binding sites in the *HNF4A* gene in mice and humans are highly conserved. Mutation experiments showed that miR34a binds to the second binding site (Figure 2 and Figure 4) in 3′-UTR. Furthermore, overexpression of the human P53 protein increased miR34a expression in human HepG2 cells, thereby downregulating *HNF4A*. In addition, 13 lipid-related genes were significantly downregulated in NASH patients compared to healthy controls. Min et al. [28] showed an increased level of hepatic HMGCR mRNA and protein in NAFLD patients, which suggest that effects on *HMGCR* are transcriptionally regulated. This may be due to increased activation of *SREBP2*, the principal activator for *HMGCR*, which seems specific for NAFLD patients as it was not shown in weight-matched obese or hepatitis C patients as controls [28]. Potential mechanisms for increased activation of *SREBP2* could be the free cellular cholesterol content—which causes cellular lipotoxicity and consequently an inflammatory response, or may activate an unfolded protein response [57]—but these mechanisms remain to be elucidated. Loyer et al. [41] showed in mouse models that *PPARA* is targeted by miR21 and that downregulation of miR21 prevents NASH development. Min et al. [28] showed that miR34a can modulate HMGCR phosphorylation and may play a role in maintaining this enzyme in its active form. Ding et al. [49] showed in FFA-laden LO2 cells (normal human hepatocyte cell line) that miR34a downregulates *PPARA* expression. Therefore, it would be interesting to explore if there is an interplay between miR21/34a and the *PPARA* target *AMPK*. Though overlap exists between mice and human data, the abundance of data from in vitro and animal studies needs to be confirmed in humans to better understand the mechanistic link between miRNAs, cholesterol metabolism and NAFLD.

To conclude, we here show that miR122, miR34a, miR21 and miR132—all highly expressed in NASH—could relate to some of the cholesterol-related pathophysiological characteristics of NAFLD. Moreover, it is of interest to explore in more detail whether adding levels of plasma miRNAs (122, 34a and 379) to current noninvasive biomarkers improves the diagnosis of NAFLD and/or its different disease stages. Finally, we also identified several mechanistic leads from animal studies that need to be confirmed in human studies.

## Figures and Tables

**Table 1:** miRNAs and their target genes.

miRNA	Target Genes
122 [24]	*CYP7A1*, *SRF*, *RAC1*, *RHOA*, *CCNG1*, *GTF2B*, *GYS1*, *NFATC2IP*, *ENTPD4*, *ANXA11*, *FOXP1*, *MECP2*, *NCAM1*, *TBX19*, *AACS*, *DUSP2*, *ATP1A2*, *MAPK11*, *AKT3*, *GALNT10*, *G6PC3*, *SLC7A1*, *FOXJ3*, *SLC7A11*, *TRIB1*, *DSTYK*, *PRKAB1*, *ACVR1C*, *PRKRA*, *PTP1B*, *P4HA1*, *ZNF395*, *SOCS1*, *HMOX1*, *CDK4*
34a [29]	*HNF4A*, *MTP*, *APOB*, *SREBP1C*, *ACC1*, *ACC2*, *HMGCR*
21 [32]	*PPARA*
21 [33]	*HMGCR*
379 [35]	Fibrosis and inflammation: *CAT*, *CTGF*, *IL10*, *PDGFA*, *PDGFRA*, *SMAD4*, *TGFBR1*, *THBS1* Energy management, including gluconeogenesis and lipogenesis: *CREB1*, *EIF4E*, *FOXO1*, *INSR*, *IGF1*, *IGF1R*, *ITPR2*, *PRKAA1*, *PRKAA2*, *RICTOR*, *SOCS1*, *TCF7L2* Cell survival and proliferation: *BCL2*, *CCNB1*, *HGF*, *PMAIP1*, *PTEN*, *YAP1* Signaling pathways: *HDAC2*
29a [24]	*DK6*, *RAN*, *BACE1*, *S100B*, *IMPDH1*, *GLUL*, *PPM1D*, *PIK3R1*, *LPL*, *CPEB3*, *CPEB4*, *ADAMTS9*, *TRIM63*, *MYCN*, *SERPINB9*, *DICER1*, *TNFAIP3*, *CDC42*, *PXDN*, *ITIH5*, *PTEN*, *ABL1*
144	Not reported
33a [36]	*CROT*
33b* [36]	*CROT*
33b	Not reported
451	Not reported
132 [37]	*ACHE*, *FOXO3*, *PTEN*, *SIRT1*
129	Not reported
486	Not reported

**Table 2:** Correlations between miRNA and NASH-related parameters.

MiR	With	Correlation	Where	Author
122	IL-1α	r = 0.250; *p* = 0.030; *n* = 75	serum	[20]
	TAG	r = 0.230; *p* = 0.048; *n* = 75	serum	[20]
	VLDL-C	r = 0.230; *p* = 0.048; *n* = 75	serum	[20]
	HDL-C	r = -0.305; *p* = 0.001; *n* = 65	serum	[21]
	ALP	r = 0.306; *p* = 0.021; *n* = 65	serum	[21]
	ALT	r = 0.351; *p* < 0.001; *n* = 65	serum	[21]
	AST	r = 0.367; *p* < 0.001; *n* = 65	serum	[21]
	Hepatocellular ballooning	r = 0.200; *p* = 0.035; *n* = 65	liver	[21]
	Lobular inflammation	r = 0.225; *p* = 0.017; *n* = 65	liver	[21]
	Liver	r = 0.253; *p* = 0.019; *n* = 65	serum	[21]
	ALT	r = 0.75; *n* = 34	serum	[22]
	AST	r = 0.55; *n* = 34	serum	[22]
	Fibrotic stage	r = 0.33; *n* = 34	serum	[22]
	Inflammation activation	r = 0.33; *n* = 34	serum	[22]
	LDL-C	r = 0.44; *n* = 34	serum	[22]
	TC	r = 0.36; *n* = 34	serum	[22]
	Fibrotic stage	r = 0.399; *p* < 0.002; *n* = 56	serum	[24]
	NAS	r = 0.306; *p* = 0.022; *n* = 56	serum	[24]
men	Severity of steatosis	normal vs. mild *p* < 0.001; *n* = 90 vs. *n* = 37	serum	[26]
		mild vs. severe *p* = 0.047; *n* = 37 vs. *n* = 11	serum	[26]
women	Severity of steatosis	normal vs. mild *p* = 0.002; *n* = 221 vs. *n* = 36	serum	[26]
		mild vs. severe *p* = 0.035; *n* = 36 vs. *n* = 8	serum	[26]
34a	Fibrotic stage	r = 0.41; *n* = 34	serum	[22]
	Inflammation activation	r = 0.43; *n* = 34	serum	[22]
	TAG	r = 0.43; *n* = 28	serum	[25]
	VLDL-C	r = 0.44; *n* = 28	serum	[25]
21	Fibrosis	r = 0.461; *p* = 0.021; *n* = 19	liver	[41]
	Hepatic ballooning	r = 0.713; *p* < 0.001; *n* = 19	liver	[41]
	Lobular inflammation	r = 0.735; *p* < 0.001; *n* = 19	liver	[41]
	Steatosis	r = 0.539; *p* = 0.005; *n* = 19	liver	[41]
379	ALP	r = 0.278; *p* = 0.048; *n* = 53	serum	[35]
	TC (all participants)	r = 0.361; *p* = 0.039; *n* = 53	serum	[35]
	LDL-C	r = 0.285; *p* = 0.043; *n* = 53	serum	[35]
	Non-HDL-C	r = 0.286; *p* = 0.038; *n* = 53	serum	[35]
	TC (non-statin users)	r = 0.381; *p* = 0.045; *n* = 42	serum	[35]
29a	TAG	r = 0.144; *p* = 0.048; *n* = 46	serum	[24]
33b *	AST	r = 0.203; *p* = 0.046; *n* = 61	serum	[21]
	HDL-C	r = -0.276; *p* = 0.004; *n* = 61	serum	[21]
	Hepatic ballooning	r = 0.343; *p* = 0.001; *n* = 13	liver	[21]
	Lobular inflammation	r = 0.358; *p* < 0.001; *n* = 12	liver	[21]
	TAG	r = 0.279; *p* = 0.004; *n* = 61	serum	[21]
33a	HDL-C	r = -0.313; *p* = 0.004; *n* = 74	serum	[36]
144	HDL-C	r = -0.221; *p* = 0.043; *n* = 74	serum	[36]
129	TAG	r = 0.662; *p* < 0.001; *n* = 117	serum	[39]
	TC	r = 0.708; *p* < 0.001; *n* = 117	serum	[39]
132	ApoE	β ± SE = 0.038 ± 0.002; *p* = 0.017; *n* = 140	serum	[38]
	ALT	β ± SE = 0.005 ± 0.002; *p* = 0.018; *n* = 140	serum	[38]
	NAFLD	OR 3.08 (1.06, 8.99); *p* = 0.0392; *n* = 140	serum	[38]
	TAG	β ± SE = 0.072 ± 0.029; *p* = 0.015; *n* = 140	serum	[38]

Abbreviations: IL-1α—Interleukin 1 alfa; TAG—triacylglycerol; VLDL-C—very-low-density lipoprotein cholesterol; HDL-C—high-density lipoprotein cholesterol; ALP—alkaline phosphatase; ALT—alanine-aminotransferase; AST—aspartate-aminotransferase; LDL-C—low-density lipoprotein cholesterol; TC—total cholesterol; NAS—NAFLD Activity Score; Non-HDL-C—non-high-density lipoprotein cholesterol; apoE—apolipoprotein E; NAFLD—non-alcoholic fatty liver disease.

**Table 3:** Receiver Operation Characteristics.

miR		AUC	Significance	Sensitivity	Specificity	PPV (%)	NPV (%)	Author
122	Hepatocellular ballooning ^a,b^	0.76		74.4%	46.8%	46.8%	87.3%	[21]
	Lobular inflammation ^a,c^	0.76		74.4%	46.8%	46.8%	87.3%	[21]
	NAFLD ^a,d^	0.82		83.1%	69.8%	47.8%	92.5%	[21]
	ALT	0.91						[22]
	NAFLD-ss ^e^	0.93						[22]
	NAFLD-ss ^e^ vs. NASH ^f^	0.70						[22]
		0.83	*p* < 0.001	75.0%	82.4%			[24]
		0.86	*p* = 0.001, 95% CI = 0.77–0.95					[25]
34a		0.78	*p* = 0.001, 95% CI = 0.66–0.90					[25]
	ALT	0.83	*p* = 0.001, 95% CI = 0.73–0.94					[25]
	NAFLD-ss ^e^ vs. NASH ^f^	0.76						[22]
379	NAFL	0.76						[35]
	NAFLD	0.72						[35]
	NASH	0.72						[35]
	Early stage NAFLD	0.74						[35]
	Advanced stage NAFLD	0.67						[35]
29a		0.68	*p* = 0.007	60.9%	82.4%			[24]
129	NAFLD	0.93		83.8%	92.7%			[39]

Abbreviations: NAFLD—non-alcoholic fatty liver disease; ALT—alanine-aminotransferase; NAFLD-ss—non-alcoholic fatty liver disease simple steatosis; NASH—non-alcoholic steatohepatitis. ^a^ optimum conditions selected. ^b^ discriminating hepato-cellular from non-hepato-cellular ballooning. ^c^ discriminating lobular inflammation from non-lobular inflammation. ^d^ discriminating NAFLD from control. ^e^ NAS score 1–4. ^f^ NAS score 5–7.
